# A New Operation for the “Radical” Cure of Hydrocele*Extract from a paper read before the Buffalo Medical Club, April 28, 1880.

**Published:** 1880-07

**Authors:** Bernard Bartow


### A NEW OPERATION FOR THE "RADICAL" CURE OF HYDROCELE.*

BY BERNARD BARTOW, M. D.

THE following operation for the so-called "radical" cure of hydrocele, I have employed in two instances, with such satisfactory results as to lead me to believe there are some points of value in the method, and particularly, in its application to cases which have resisted the means ordinarily employed for the relief of this disease. The operation consists of an incision from three to four inches in length, in the scrotum—in the centre of the hydrocele tumor,—extending through the scrotal subcutaneous tissues until the sac is exposed. The loose connective tissue is then separated from the sac to the extent of about an inch either side of the line of the incision, exposing about one-third the circumference of the tumor—the distended sac protruding into the wound, renders this last step very easy of accomplishment. Into the most depending part of the tumor thus exposed, a fine trochar and canula is introduced, and the fluid is drawn off; the entire wound being left to close by granulation. It is intended that air shall not be admitted into the sac; and it is preferable to make the incision with antiseptic precautions, and to continue them during its subsequent treatment. In the two cases where this plan was used, the first was a large hydrocele that had received no previous treatment, the second case being one in which repeated tapping had been performed; both patients were young married men between thirty and thirty-five years of age. The clinical features following the operation were very similar to those following the injection of the sac with tr. iodine. In both instances the sac had refilled by the fourth day. Resorption was complete by the tenth day in case 1; in case 2, however, I did not wait for this event to follow, but re-tapped the sac through the wound on the sixth day, after which it did not re-fill. The degree of inflammation in the scrotal sub-cutaneous tissue and sac was quite active in the first case, but

*Extract from a paper read before the Buffalo Medical Club, April 28, 1880.

the free incision of the operation prevented any tension in the part, and there was no sloughing of scrotal tissue, or any other untoward feature. On this occasion no special antiseptic measures were observed.

In the second case, however, strict antiseptic precautions were employed throughout, with the effect to confine the inflammatory action within very moderate limits. I was strongly impressed with the influence antisepsis exerted in subduing the subsequent inflammation, by the fact, that in this instance the dissection of connective tissue from the sac was much greater than in the first case, and this, without some modifying agent, would have resulted in a much greater degree of inflammatory action in the part. The extent of constitutional disturbance was indicated by a rise in patient's temperature of 1° above normal, and the local appearances were such as indicated a slight but general implication of the entire sac and surrounding tissues in the inflammatory process.

In the first case the patient was kept quiet until the tenth day, while patient No. 2 was confined to his bed for one day only—that following the operation. In both cases the scrotum was supported by a suspensory during the time the incision was healing, which latter was complete by the fourteenth day. At the end of nine months there was no sign of the disease returning in case 1, while in case 2 the sac had not refilled during the period of four months that it was under observation. Following the operation in both cases, the testicle was movable in its sac, showing that obliteration of the sac did not take place.

The idea upon which this operation is based, is that of identity and continuity of the connective tissue composing the sac, with the less dense connective tissue which would be described as lying *outside* the sac; and that by exciting inflammatory action in this *outside* connective tissue, it will extend to and involve that composing the sac, by continuity of structure. By wounding and disturbing the parts in close relation to the sac, we thereby apply the irritant upon its outer surface, and by the resulting inflammation induce those changes in the vascular system of the part, upon which would seem to depend the restoration of the normal secretion of the tunica vaginalis. Admitting that the changes resulting from the inflammation principally affect the vessels supplying the part, it would seem by this method that we could quickly and with certainty induce those changes, by acting thus directly upon the tissue in which the vessels are imbedded.

In view of the fact that there are a considerable number of cases of hydrocele, in which injection with tr. iodine fails to accomplish a cure, and that these cases (if they obtain relief) become subjects for more objectionable and severe operations, I think there are advantages in the method here advanced, that will recommend it as a substitute for either the seton or the operation of incising the sac—the method usually employed after failure with tr. iodine injection. It is free from the dangerous constitutional disturbance liable to follow inflammation in an open serous sac—as in the case where a hydrocele is incised; and the prolonged suppuration attending obliteration of the sac by incision, is superceded by that which would follow from a superficial wound only. By preventing access of air to the interior of the sac, the liability to suppuration within the sac is almost nil; this principal danger being avoided, the method would seem to possess the conditions by which inflammation could be excited with safety in the sac and surrounding tissues.

